# pSTAT3 transactivates EGFR in maintaining EGFR protein homeostasis and EGFR-TKI resistance: pSTAT3 transactivates EGFR in TKI resistance

**DOI:** 10.3724/abbs.2024166

**Published:** 2024-09-29

**Authors:** Juan Ao, Junjie Fei, Guoqiang Wang, Wenhua Zhang, Shuhan Yu, Rongtian Guo, Mengmeng Niu, Hu Chen, Yang Cao, Zhi-Xiong Jim Xiao, Yong Yi

**Affiliations:** 1 Center of Growth Metabolism and Aging Key Laboratory of Bio-Resource and Eco-Environment Ministry of Education College of Life Sciences Sichuan University Chengdu 610064 China; 2 Department of Cardiothoracic Surgery the First Affiliated Hospital of Chengdu Medical College Chengdu 610500 China

**Keywords:** EGFR, pSTAT3, protein homeostasis, EGFR-TKI resistance

## Abstract

EGFR protein trafficking is critical for regulating multiple biological processes, including cell growth and survival. However, how EGFR protein homeostasis is maintained remains unclear. In this study, we show that a reduction in plasma membrane-associated EGFR triggers EGFR transcription by promoting pSTAT3 nuclear localization. Nucleus-localized pSTAT3 binds to the EGFR gene promoter to transactivate EGFR. Moreover, erlotinib, an EGFR tyrosine kinase inhibitor (TKI), can also increase pSTAT3 nuclear accumulation, resulting in increased EGFR transcription and erlotinib resistance. Importantly, pharmacological inhibition of pSTAT3 can significantly overcome the resistance of cancer cells to erlotinib. Together, these findings demonstrate that pSTAT3 is pivotal for maintaining EGFR protein homeostasis and suggest that activation of the pSTAT3-EGFR axis contributes to EGFR-TKI resistance.

## Introduction

Epidermal growth factor receptor (EGFR) plays pivotal roles in multiple biological processes, including cell proliferation, migration, and survival [ 1– 4]. Abnormal activation of the EGFR signaling pathway serves as a driving force in lung cancer development [ 4, 5]. EGFR gene amplification, deletion, and point mutation can lead to abnormal EGFR activation [ 6, 7]. L858R mutation and exon 19 deletion are the most common EGFR-activating mutations in non-small cell lung cancer (NSCLC) patients [ 4, 8, 9]. The specific targeting of EGFR-activating mutations by 1st-generation EGFR tyrosine kinase inhibitors (TKIs), such as erlotinib and gefitinib, is beneficial for NSCLC patients [ 8, 10]. However, most NSCLC patients develop acquired resistance to 1st-generation TKIs within 10-16 months, in which the EGFR T790M mutation accounts for approximately 50% of all 1st-generation EGFR TKI resistance [ 9, 11]. Moreover, HER2 amplification, MET amplification, BRAF mutation, PIK3CA mutation, Ras mutation, or activation of IGF1R also contribute to EGFR TKI resistance [ 12– 15].

In addition to gene mutation(s), increased EGFR protein expression plays a critical role in regulating cancer development [6].The upregulation of EGFR protein recycling, stability, or gene transcription can facilitate EGFR protein expression [6]. A series of stress signals, including serum starvation, UV irradiation, hypoxia, oxidative stress, and erlotinib/gefitinib treatment, can inhibit EGFR recycling to the plasma membrane and consequently lead to EGFR protein degradation [16]. It has been reported that the ubiquitin E3 ligase c-Cbl can facilitate EGFR mono-ubiquitination, which leads to EGFR lysosome-dependent degradation [17]. Moreover, several E3 ubiquitin ligases, including FBXL2, CHIP, HUWE1, Siah1, and CGRRF1, can promote EGFR poly-ubiquitination and result in the proteasomal degradation of EGFR [ 18– 22]. With respect to EGFR gene transcription, several transcription factors, such as Sp1, c-Jun, and Stat5b, can directly transactivate EGFR [ 23– 25]. However, the role of the balance between EGFR protein degradation and gene transcription in maintaining EGFR protein homeostasis to adapt to stress signaling remains unknown.

In this study, we show that a reduction in plasma membrane-associated EGFR expression promotes EGFR transcription in a pSTAT3-dependent manner to maintain EGFR protein homeostasis. Moreover, erlotinib promoted pSTAT3-mediated EGFR transcription, which resulted in resistance to erlotinib in NSCLC cells. Together, the results of this study highlight that pSTAT3 directly transactivates EGFR, which plays a critical role in maintaining EGFR protein homeostasis, and that the activation of pSTAT3-EGFR signaling is a novel molecular mechanism by which NSCLC cells develop acquired resistance to EGFR TKIs.

## Materials and Methods

### Cell culture and drug treatments

A549, H1975, PC-9, H1299, and HEK 293T cells were obtained from the American Type Culture Collection (ATCC, Manassas, USA). A549, H1299, and HEK 293T cells were cultured in DMEM (GIBCO, Rockville, USA) supplemented with 10% fetal bovine serum (FBS; HyClone, Logan, USA). H1975 and PC-9 cells were cultured in RPMI-1640 medium (GIBCO) supplemented with 10% FBS. All the cells were grown in media supplemented with 100 units/mL penicillin (GIBCO) and 100 μg/mL streptomycin (GIBCO). The cells were maintained in a humidified 37°C incubator under a 5% CO _2_ atmosphere. The cells at 75–85% confluence were treated with the indicated chemical compound(s), as indicated. 2-deoxy-D-glucose (2-DG; D8375) was purchased from Sigma (St Louis, USA). Ruxolitinib (S1378), actinomycin D (S8964), and erlotinib (S7786) were purchased from Selleck (Houston, USA). Recombinant Human EGF (236-EG) was purchased from R&D systems (Minnesota, USA).

### Plasmid transfection, lentiviral infection, and RNA interference

Cells at 80% confluence were transfected via Lipofectamine 2000 (Invitrogen, Carlsbad, USA). Lentiviruses were amplified via the transfection of HEK293T cells with psPAX2 and pMD2.G packaging plasmids and lentiviral-based plasmids via Lipofectamine 2000. The viruses were collected at 60 h after transfection. The cells at 50% confluence in the presence of 10 μg/mL polybrene (C0351; Beyotime, Shanghai, China) were infected with recombinant lentivirus, followed by 12 h of incubation at 37°C with 5% CO _2_. Lentivirus-based shRNAs specific for green fluorescent protein (GFP, 5'-GAAGCAGCACGACTTCTTC-3') or EGFR (5'-CGCAAAGTGTGTAACGGAATA-3') were constructed, with the sequences synthesized by Sangon Biotech (Chengdu, China).

### Western blot analysis

The cells were collected, washed with PBS, and resuspended in EBC _250_ lysis buffer (250 mM NaCl, 50 mM Tris pH 8.0, 0.5% Nonidet P-40, 50 mM NaF, 1 mM phenylmethylsulfonyl fluoride, 2 μg/mL aprotinin, and 2 μg/mL leupeptin). Equal amounts of protein were separated by SDS-PAGE, transferred to PVDF membranes (Millipore, Billerica, USA), and hybridized to an appropriate primary antibody and HRP-conjugated secondary antibody for subsequent detection via enhanced chemiluminescence. Antibodies against actin (sc-1615) and SP1 (sc-59) were purchased from Santa Cruz Biotech (Santa Cruz, USA). Antibodies against EGFR (4267), pAKT (4508), AKT (9272), and pSTAT3 (9134) were purchased from Cell Signaling Technology (Danvers, USA). Antibody against GAPDH (AB0037) was purchased from Abways (Shanghai, China).

### Immunofluorescence analysis

The cells were cultured in a 12-well plate with a coverslip, fixed in 4% paraformaldehyde, washed with PBS, permeabilized in 0.3% Triton X-100, washed with PBS, hybridized to an EGFR (1:200, 4267; Cell Signaling Technology) or pSTAT3 (1:100, 9134; Cell Signaling Technology) antibody, incubated with a FITC-conjugated secondary antibody (Jackson ImmunoRsearch, Westgrove, USA) and counterstained with DAPI (Beyotime) for subsequent detection. Coverslips were mounted with ProLong Gold antifade reagent (Invitrogen). Images were acquired via the Leica TCS SP5 II system. To determine the co-localization, the free software Image J. Fiji coupled with the Coloc 2 plugin and Pearson’s correlation coefficient were used to calculate double fluorescence correlation coefficients, and co-localized fluorescence quantifications were presented by scatter diagrams.

### Cell viability assay

Cell viability assay was performed using a CellTiter_96 kit (Promega, Madison, USA) as described in the manufacture’s instruction. Briefly, 5 × 10 ^3^ cells were seeded into a 96-well plate and cultured overnight and then treated with the indicated chemical compound for 72 h. After incubation, 10 μL of MTS reagent was added to each well and incubated for 1 h to allow metabolically active cells to convert MTS into a formazan product. Absorbance was measured at 490 nm using a microplate reader (VARIOSKAN FLASH; Thermo Fisher Scientific, Waltham, USA) to assess cell viability.

### Quantitative PCR (qPCR)

Total RNA was extracted from cells via an RNA easy Plus Mini Kit (Qiagen, Hilden, Germany) according to the manufacturer’s protocol. The RNA was reverse-transcribed into cDNA via an M-MLV First Strand Kit (Invitrogen). qPCR were performed in a CFX96 Real-Time PCR System (Bio-Rad, Hercules, USA) via SoFast EvaGreen Supermix (Bio-Rad) according to the manufacturer’s instructions. The reactions were carried out in a 96-well plate at 95°C for 5 min, followed by 40 cycles of 95°C for 15s and 60°C for 30 s. The primers used are *EGFR*: (F) 5′-TCCAGGAGGTGGCTGGTTAT-3′, (R) 5′-TGCAGGTTTTCCAAAGGAATTC-3′ and *Actin* (F) 5′-TCACTATTGGCAACGAGCGGTTC-3′, (R) 5′-GCACTGTGTTGGCATAGAGGTCTT-3′. Actin expression was used as an endogenous control to normalize EGFR gene expression via the ΔΔCt method.

### Chromatin immunoprecipitation (ChIP) assay

ChIP assays were performed in PC-9/DR cells with a ChIP-IT Kit (Active Motif, Carlsbad, USA) using antibodies specific for pSTAT3 (Cell Signaling Technology) or normal rabbit IgG (Invitrogen). The ChIP samples were subjected to PCR experiments to amplify fragments of the *EGFR* promoter elements via two pairs of primers (P1 F: 5'-GCACAGATTTGGCTCGACCT-3'; R: 5'-CGGGTGCCCTGAGGAGTTAA-3'; P2 F: 5'-GTCCCATGAAGATGTTCAGC-3'; R: 5'-AGCCTTCATAGTACGGCTTG-3'). To examine the strength of pSTAT3 binding to EGFR promoter elements, ChIP samples were subjected to qPCR or reverse transcription PCR using the indicated primers. The value of each ChIP sample was normalized to its corresponding input.

### Cellular fractionation

Cellular fractionation of cytoplasm and nucleus was performed as described in the Nuclear and Cytoplasmic Protein Extraction Kit (P0028; Beyotime). Briefly, cells were collected, washed twice with cold PBS, and resuspended in cytosolic lysis buffer. The mixture was vortexed vigorously at the highest speed for 5 s to completely suspend and disperse the cell precipitate, and put in an ice bath for 10‒15 min. After centrifugation (12,000 *g*, 5 min, 4°C), the cytoplasm fraction in the supernatant was collected. Then nuclear lysis buffer was added into the precipitation and vortexed strongly for 30 min. After centrifugation (12,000 *g*, 5 min, 4°C) the nuclear fraction was collected.

### Statistical analyses

GraphPad Prism 8.0 (GraphPad Software, Inc., La Jolla, USA) was used for data recording, collection, processing, and calculation. All the experiments were performed at least two times. Data are presented as the mean ± SD. Quantitative data were analyzed statistically via Student’s *t* test to assess significance.

## Results

### Downregulation of plasma membrane-associated EGFR facilitates EGFR transcription

**Figure 1:**
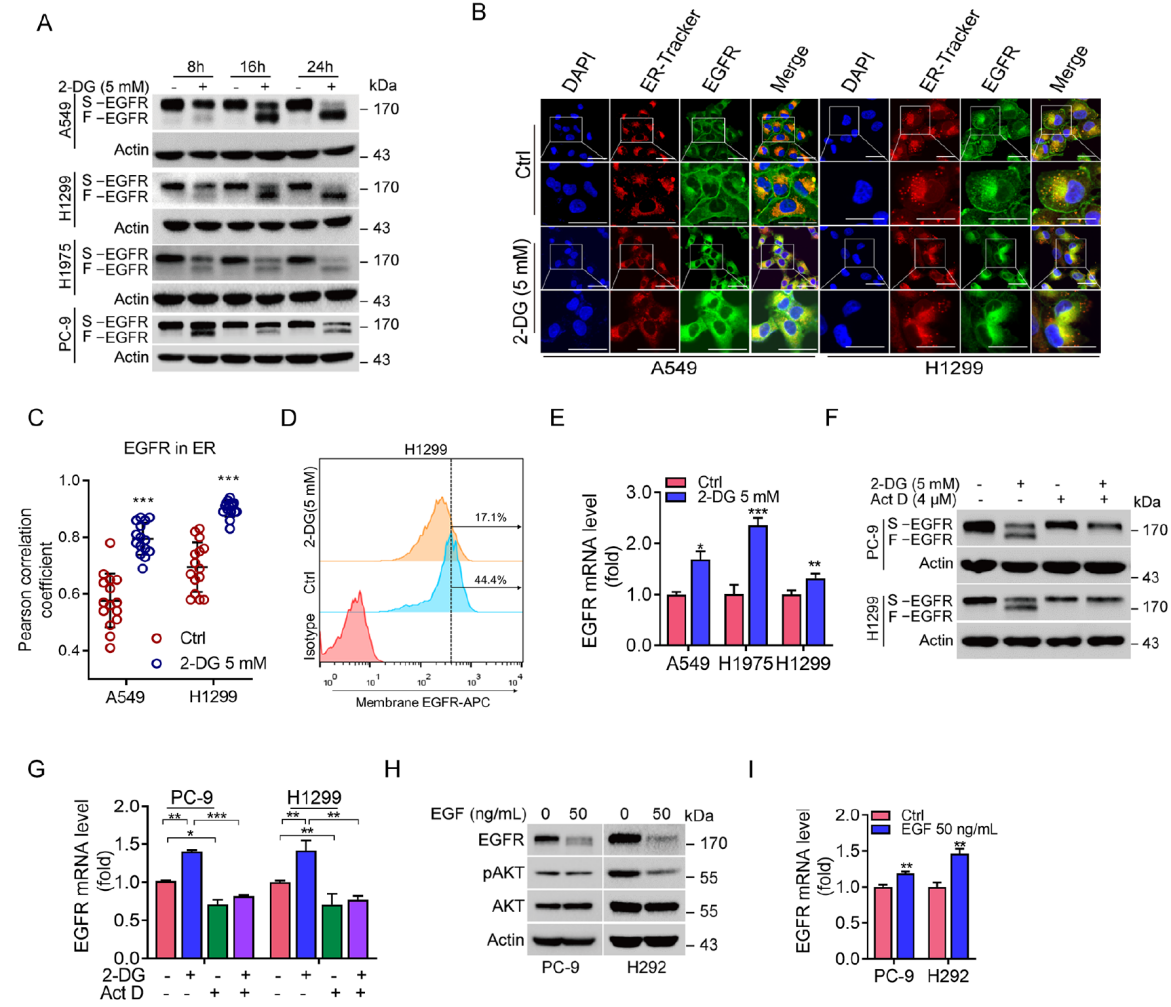
Figure 1 A reduction in plasma membrane-associated EGFR protein expression triggers *de novo* EGFR transcription (A) A549, H1299, H1975, or PC-9 cells were treated with 5 mM 2-DG for the indicated durations. The cells were subjected to western blot analysis. (B,C) A549 or H1299 cells were treated with or without 5 mM 2-DG for 24 h. The cells were subjected to immunofluorescent analysis (B). Scale bar: 25 μm. The co-localization between EGFR and ER-Tracker (as analyzed by Pearson’s correlation coefficient) was quantified and statistically analyzed. Fifteen cells derived from two independent experiments were randomly chosen and subjected to quantification analysis (C). (D) H1299 cells were treated with or without 5 mM 2-DG for 24 h. Cells were subjected to FACS analysis for cell surface EGFR expression. (E) A549, H1975, or H1299 cells were treated with or without 5 mM 2-DG for 24 h. The cells were subjected to qPCR analysis. (F,G) PC-9 or H1299 cells were treated with or without 5 mM 2-DG in the presence or absence of 4 μM actinomycin D (Act D) for 20 h. The cells were subjected to western blot analysis (F) or qPCR (G). (H,I) PC-9 or H292 cells were grown in serum-free medium for 12 h prior to treatment with 50 ng/mL EGF for 5 h. The cells were subjected to western blot analysis (H) or qPCR (I). Data are presented as the mean ± SD. *P < 0.05; **P < 0.01; ***P < 0.001.

EGFR trafficking is critically important for maintaining EGFR protein homeostasis through its recycling to the plasma membrane or to lysosomes for protein degradation [6]. However, how EGFR protein homeostasis is maintained remains largely unknown. Since EGFR trafficking requires post-translational modification of EGFR, including glycosylation [26], we first employed 2-DG, an inhibitor of glycolysis, to block protein glycosylation in the endoplasmic reticulum (ER) [27]. As shown in Figure 1A, treatment of non-small cell lung cancer cells (A549, H1299, H1975, and PC-9) with 2-DG led to the appearance of two EGFR bands on SDS-PAGE, a slower migrated EGFR (S-EGFR) band and a fast migrated EGFR (F-EGFR) band. Notably, treatment with 2-DG significantly reduced S-EGFR protein expression concomitant with increased F-EGFR expression ( Figure 1A). Immunofluorescence analyses revealed that treatment with 2-DG dramatically reduced plasma membrane-associated EGFR expression, concomitant with increased endoplasmic reticulum (ER)-associated EGFR expression ( Figure 1B,C). Similarly, FACS analyses also showed that 2-DG markedly downregulated cell surface EGFR expression ( Figure 1D). These results suggest that 2-DG treatment results in both the downregulation of plasma membrane-associated glycosylated EGFR (S-EGFR) expression and the upregulation of non-glycosylated EGFR expression (F-EGFR), which likely reflects newly synthesized EGFR protein. Indeed, qPCR analyses indicated that 2-DG significantly upregulated EGFR mRNA expression ( Figure 1E). Importantly, treatment with actinomycin D (Act D), a common transcriptional inhibitor, completely inhibited 2-DG-induced EGFR mRNA expression and F-EGFR protein expression ( Figure 1F,G). These results provide strong evidence that 2-DG can trigger *de novo* EGFR transcription. Therefore, we hypothesized that downregulation of plasma membrane-associated EGFR expression promotes EGFR transcription. To test this hypothesis, we treated cells with EGF, a ligand of EGFR that can rapidly induce plasma membrane-associated EGFR degradation in a lysosome-dependent manner [17]. As shown in Figure 1H,I, EGF treatment significantly reduced EGFR protein expression, concomitant with a marked increase in EGFR mRNA level. Together, these results indicate that a reduction in plasma membrane-associated EGFR facilitates EGFR transcription.

### Reduction in the plasma membrane-associated EGFR protein facilitates pSTAT3 nuclear localization to promote EGFR transcription

**Figure 2:**
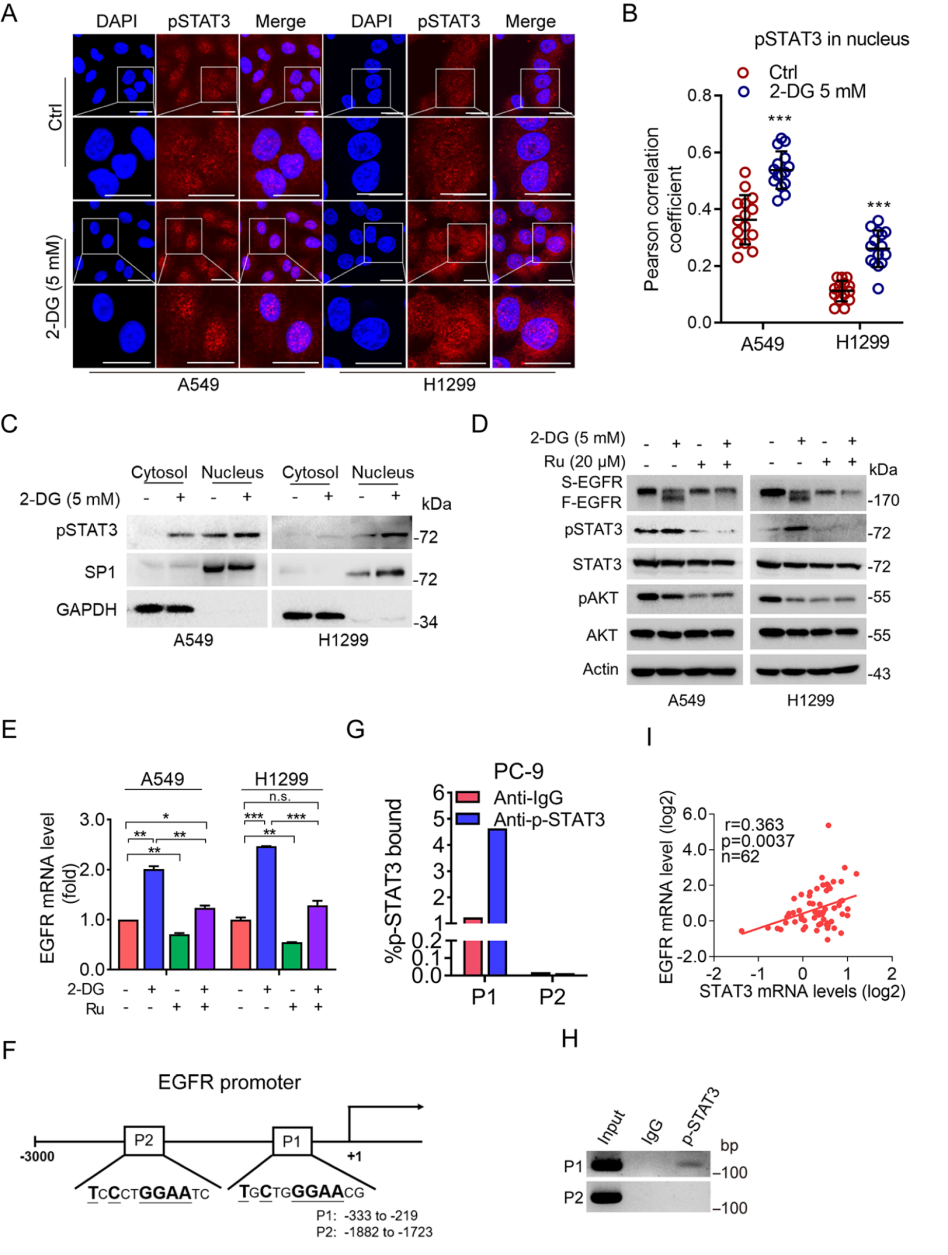
Figure 2 A reduction in the plasma membrane-associated EGFR protein facilitates pSTAT3 nuclear localization to promote EGFR transcription (A,B) A549 or H1299 cells were treated with or without 5 mM 2-DG for 24 h. The cells were subjected to immunofluorescent analysis (A). Scale bar: 25 μm. The co-localization between EGFR and ER-Tracker was quantified and statistically analyzed. Fifteen cells derived from two independent experiments were randomly chosen and subjected to quantification analysis (B). (C) A549 or H1299 cells were treated with or without 5 mM 2-DG for 24 h followed by cell fractionation and western blot analysis. (D,E) Inhibition of pSTAT3 suppresses 2-DG-induced EGFR transcription. A549 or H1299 cells were treated with or without 5 mM 2-DG in the presence or absence of 20 μM ruxolitinib (Ru) for 24 h. The cells were subjected to western blot analysis (D) or qPCR (E). (F,H) pSTAT3 directly binds to the EGFR gene promoter. A schematic diagram showing two putative pSTAT3-binding elements (P1 and P2) in the EGFR gene promoter (–1 to –3000) (F). Chromatin immunoprecipitation (ChIP) analyses were performed using a specific pSTAT3 antibody or a control IgG. Data derived from qPCR analyses (G) or reverse transcription-PCR (RT-PCR) (H) are shown. (I) Clinical correlation of STAT3 and EGFR mRNA expression in lung cancer. The Oncomine Talbot Lung dataset was analyzed for the correlation of gene expression between EGFR and STAT3. Data are presented as the mean ± SD. *P < 0.05; **P < 0.01; ***P < 0.001; n.s., not significant.

We next investigated the molecular basis by which the downregulation of plasma membrane-associated EGFR promotes EGFR transcription. Erlotinib, an EGFR-targeted tyrosine kinase inhibitor (TKI) used to treat NSCLC patients, can activate STAT3 signaling [28]. On the other hand, inhibition of STAT3 abrogates erlotinib resistance in lung cancer cells [28], suggesting a feedback loop between STAT3 and EGFR. Therefore, we investigated whether STAT3 plays a role in the regulation of EGFR transcription upon 2-DG treatment. As shown in Figure 2A,B, 2-DG significantly increased phosphorylated STAT3 (pSTAT3) in the nucleus as evidenced by immunofluorescent analyses. Moreover, cell fractionation analyses also showed that 2-DG upregulated nuclear pSTAT3 level ( Figure 2C). Importantly, treatment with ruxolitinib (Ru), a pSTAT3 inhibitor, potently suppressed the 2-DG-induced upregulation of EGFR mRNA and F-EGFR expression ( Figure 2D,E). Nuclear pSTAT3 is a transcription factor that regulates the transcription of a series of targeted genes, including *MYC*, *CCND1*, and *VEGF* [ 29– 31]. We hypothesized that pSTAT3 may directly transactivate EGFR. Indeed, we found that there are two putative pSTAT3-binding elements (TNCNNGGAA) [32] in the *EGFR* gene promoter ( Figure 2F). ChIP analyses revealed that pSTAT3 could bind to the *EGFR* promoter P1 region (–333 to –219) ( Figure 2G,H). Moreover, clinical analyses revealed a positive correlation between STAT3 and EGFR mRNA levels in lung cancer ( Figure 2I). Together, these results suggest that EGFR is a bona fide downstream transcription target of pSTAT3 and down-regulation of plasma membrane-associated EGFR facilitates pSTAT3 nuclear localization, which, in turn, promotes EGFR transcription. This feedback loop is likely important in maintaining EGFR protein homeostasis.

### Activation of the pSTAT3-EGFR axis promotes EGFR-TKI resistance in NSCLC cells

**Figure 3:**
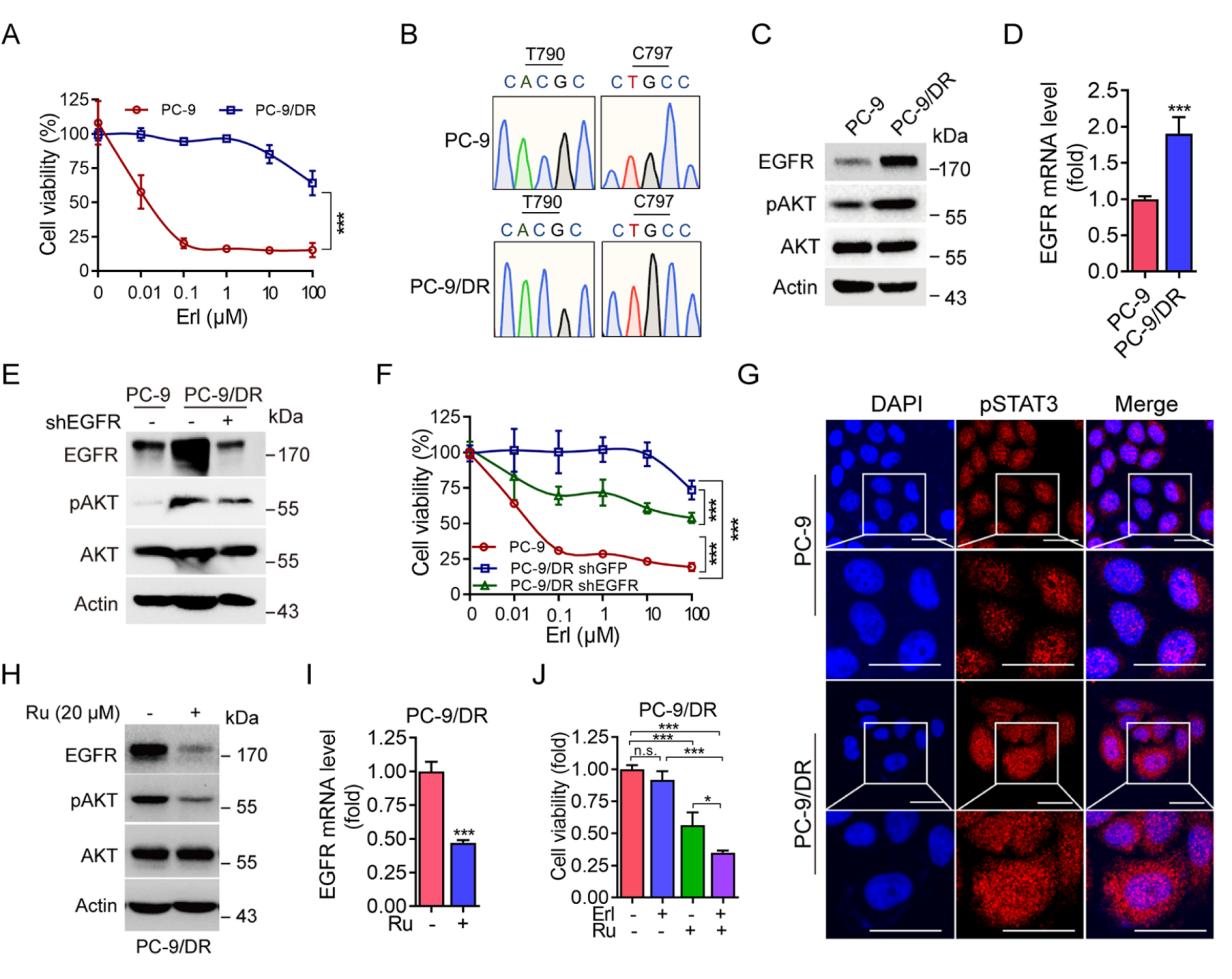
Figure 3 Activation of the pSTAT3-EGFR axis promotes resistance to EGFR-TKIs in NSCLC cells (A) PC-9 cells or PC-9 erlotinib-resistant cells (PC-9/DR) were treated with the indicated concentration of erlotinib (Erl) for 72 h. Cell viability was determined via MTS assay. (B,D) PC-9 or PC-9/DR cells were subjected to mRNA extraction, PCR for the EGFR coding sequence, and Sanger sequencing (B), western blot analysis (C), or qPCR (D). (E,F) PC-9/DR cells stably expressing shEGFR were subjected to western blot analysis (E) or treated with the indicated concentration of erlotinib (Erl) for 72 h, after which cell viability was determined via MTS assay (F). (G) PC-9 or PC-9/DR cells were subjected to immunofluorescent analysis. Scale bar: 25 μm. (H,I) PC-9/DR cells were treated with or without ruxolitinib (Ru, 20 μM) for 24 h. The cells were subjected to western blot analysis (H) or qPCR (I). (J) PC-9/DR cells were treated with or without erlotinib (10 nM) in the presence or absence of Ru (20 μM) for 4 days. Cell viability was determined via MTS assay. Data are presented as the mean ± SD. *P < 0.05; ***P < 0.001; n.s., not significant.

Our abovementioned data indicate that the inhibition of plasma membrane-associated EGFR promotes EGFR transcription in a pSTAT3-dependent manner. EGFR-TKIs, including erlotinib and gefitinib, can induce plasma membrane-associated EGFR protein degradation [16], and the inhibition of STAT3 abrogates erlotinib resistance in lung cancer cells [28], suggesting that a high level of plasma membrane-associated EGFR may be important in EGFR-TKI resistance. Therefore, we hypothesized that activated pSTAT3-EGFR signaling is a novel molecular mechanism of EGFR-TKIs resistance. To conform this hypothesis, we established spontaneous erlotinib-resistant PC-9 cells (PC-9/DR) upon treatment with a low dose of erlotinib. As shown in Figure 3A, PC-9/DR cells also exhibited strong resistance to erlotinib. Sanger sequencing revealed that PC-9/DR cells had no acquired the EGFR T790M or C797S (resistance to osimertinib) [22] mutation ( Figure 3B), indicating that the resistance of PC-9/DR cells to erlotinib cannot be attributed to the EGFR ^T790M^ mutation. Notably, compared with parental PC-9 cells, PC-9/DR cells presented significantly elevated EGFR protein and mRNA levels ( Figure 3C,D). Importantly, the silencing of *EGFR* significantly reduced PC-9/DR cell resistance to erlotinib ( Figure 3E,F). These results support the notion that high EGFR expression level plays a role in the resistance to erlotinib in lung cancer cells.

We further examined the nuclear localization of pSTAT3 in PC-9/DR cells. As shown in Figure 3G, nuclear pSTAT3 staining was significantly elevated in PC-9/DR cells. Importantly, the inhibition of pSTAT3 by ruxolitinib not only significantly reduced EGFR mRNA and protein levels in PC-9/DR cells but also sensitized PC-9/DR cells to erlotinib ( Figure 3H–J).

Taken together, our results show for the first time that activation of the pSTAT3-EGFR axis is a novel molecular mechanism by which lung cancer cells acquire resistance to EGFR-TKIs.

## Discussion

**Figure 4:**
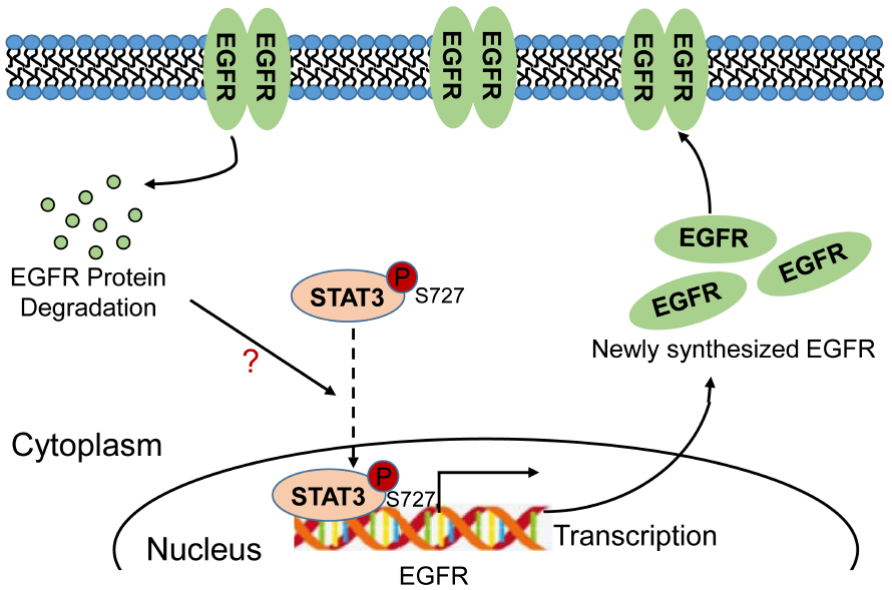
Figure 4 A model depicting the regulation of EGFR protein homeostasis Downregulation of plasma membrane-associated EGFR promotes the entry of pSTAT3 into the nucleus, which in turn transactivates EGFR, thus contributing to maintaining EGFR protein homeostasis.

Protein homeostasis is essential for all physiological processes of an organism and involves maintaining the correct concentration, subcellular location, and conformation of proteins. Plasma membrane-associated proteins, such as GLUT, Integrins, and EGFR, play critical roles in integrating extracellular signals to execute biological processes, such as cell metabolism, migration, division, and proliferation [ 6, 26, 33]. Moreover, plasma membrane-associated protein trafficking also plays an important role in regulating a series of biological processes [ 6, 26, 33]. It has been reported that plasma membrane-associated protein trafficking leads to partial protein recycling to the plasma membrane or to lysosomes for protein degradation [16]. However, how cells maintain plasma membrane-associated protein homeostasis in the trafficking process remains unclear. Here, we show that downregulation of plasma membrane-associated EGFR protein expression triggers EGFR transcription in a pSTAT3-dependent manner to maintain EGFR protein homeostasis ( Figure 4).

In this study, we show that pSTAT3 is a novel transcription factor of EGFR and that a reduction in plasma membrane-associated EGFR enhances pSTAT3 nuclear localization, suggesting that pSTAT3 is a bridge that links plasma membrane-associated EGFR protein level to EGFR gene transcription. However, how a reduction in plasma membrane-associated EGFR facilitates pSTAT3 nuclear accumulation needs to be further investigated. Moreover, activation of EGFR can increase pSTAT3 level [34], indicating that there is a positive feedback loop between EGFR and pSTAT3, which contributes to tumor malignancy.

EGFR-TKI resistance is a major challenge for the treatment of NSCLC patients. Clinical evidence indicates that the EGFR T790M mutation accounts for approximately 50% of all 1st-generation EGFR TKI (erlotinib and gefitinib) resistance [11]. Osimertinib has been developed to overcome EGFR T790M-mediated TKI resistance. However, new acquired EGFR-resistant mutations to osimertinib, including EGFR ^C797S^ and EGFR ^L718Q^, have also recently emerged [ 35, 36]. Here, erlotinib-resistant NSCLC cells without the T790M mutation presented elevated EGFR expression. Silencing of *EGFR* significantly sensitizes NSCLC cells to erlotinib, suggesting that increased EGFR expression also contributes to NSCLC cell TKI resistance in addition to acquired EGFR resistance mutations. Importantly, erlotinib-resistant NSCLC cells also presented increased pSTAT3 nuclear accumulation. Inhibition of pSTAT3 significantly inhibits EGFR expression in erlotinib-resistant NSCLC cells and resensitizes erlotinib-resistant NSCLC cells to erlotinib. Therefore, our study indicates that pSTAT3-mediated EGFR transcription is a novel molecular mechanism by which lung cancer cells are resistant to EGFR TKIs.
